# Toward interchangeable biologics

**DOI:** 10.1002/cpt.39

**Published:** 2015-01-21

**Authors:** M McCamish, J Pakulski, C Sattler, G Woollett

**Affiliations:** ^1^ Department of Biopharmaceutical & Oncology Injectables Development Sandoz International GmbH Holzkirchen Germany; ^2^ U.S. Biopharmaceutical Regulatory Affairs, Sandoz, a Novartis Company Princeton New Jersey USA; ^3^ U.S. Clinical Development and Medical Affairs, Biopharmaceuticals North America, Sandoz, a Novartis Company Princeton New Jersey USA; ^4^ Avalere Health Washington DC USA

## Abstract

A biosimilar is designed to match the reference product – to be as close to the reference as the reference is to itself considering batch‐to‐batch variability and manufacturing changes over its lifetime.^1^ Interchangeability will require additional data, however, the interchangeable biologic itself will be the same as that approved for biosimilarity.

## BACKGROUND

There is a single regulatory standard in the United States for all biologics licensed under the Public Health Service Act, namely safety, purity, and potency. This applies to biosimilars too.^2^ Similarly, the quality of a biologic does not change based on the regulatory pathway chosen by the sponsor.

With simple generic drugs and complex generics, including generic biologics like enoxaparin (where the product is a biologic in science but regulated as a drug under the Federal Food, Drug, and Cosmetic Act), the goal is to make the “same” product. However, in the case of enoxaparin, a determination of “sameness” does not indicate an identical active molecular ingredient given that the reference product is a complex mixture of linear sugar molecules. Importantly, these complex biological products are approved by the Food and Drug Administration (FDA) as fully substitutable, meaning that they are interchangeable with the reference product for all conditions of use.

Science supports that biosimilars can be approved as interchangeable too. This requires addressing analytical, functional, and clinical biosimilarity as is done in the initial evaluation of biosimilarity. The additional hurdle of interchangeability involves providing evidence that switching back and forth with the reference product does not result in exaggerated or clinically relevant immunogenicity.

Extensive scientific progress has been made since the first biologics were licensed in the early 20th century. There is now considerable experience with the originator biologics, including making manufacturing changes to scale up or modernize manufacturing processes. Creation of biosimilars applies a similar scientific and regulatory concept. The European Medicines Agency is comfortable calling both approaches comparability, whereas the FDA distinguishes the two. Nonetheless, both settings invoke the “highly similar” analytical standard for the two products that are being compared (biosimilar to reference vs. pre to post‐manufacturing change products), and both require an increasingly comprehensive understanding of structure‐function relationships in order for the determination of “no clinically meaningful differences” to be accepted absent complete clinical studies in every indication. Immunogenicity studies are an additional consideration for biosimilars and particularly “interchangeable” biologics although generally not required before a manufacturing change.

Enoxaparin is used in critical care indications with lethal consequence if the product does not work. In approving enoxaparin as a fully substitutable complex generic drug of biologic origin in 2010, the FDA identified five criteria for addressing “sameness” in lieu of comparative clinical trials (including physicochemical attributes and fragmentation methods; sourcing; nature and arrangement of components; anticoagulant assays, and human responses).^3^ Biosimilars utilize a different regulatory pathway (351(k)), but ultimately approval and interchangeability requires the same confidence that the biosimilar has the “same” active pharmaceutical ingredient as the reference product, and can be switched without impact on the patient. FDA guidance on interchangeability is not yet available. Data expected will likely include “switching studies” in patients, while monitoring immunogenicity, demonstrating no difference compared to no switching.

## DEVELOPMENT OF A BIOSIMILAR/INTERCHANGEABLE BIOLOGIC

### Analytical studies provide the basis for a determination of biosimilarity

The “design space” for a biosimilar is created by the biosimilar sponsor's in‐depth analysis of multiple lots of their chosen reference product. This provides the specifications for the biosimilar and the justification for clinical acceptability when the biosimilar product attributes fall within the ranges of each analytical attribute of the reference product.

On the basis of the “high similarity” 4 shown by these studies, the FDA and the sponsor agree the extent of the clinical program needed to confirm biosimilarity. For biosimilars, it is critical to have a tailored clinical program that simply confirms similarity, not excessive clinical trials to re‐prove safety and efficacy of the original molecule. This enables control of development costs without compromise in quality, and is essential to the commercial viability of biosimilars and the increased access and affordability they will give to patients.

**Figure 1:**
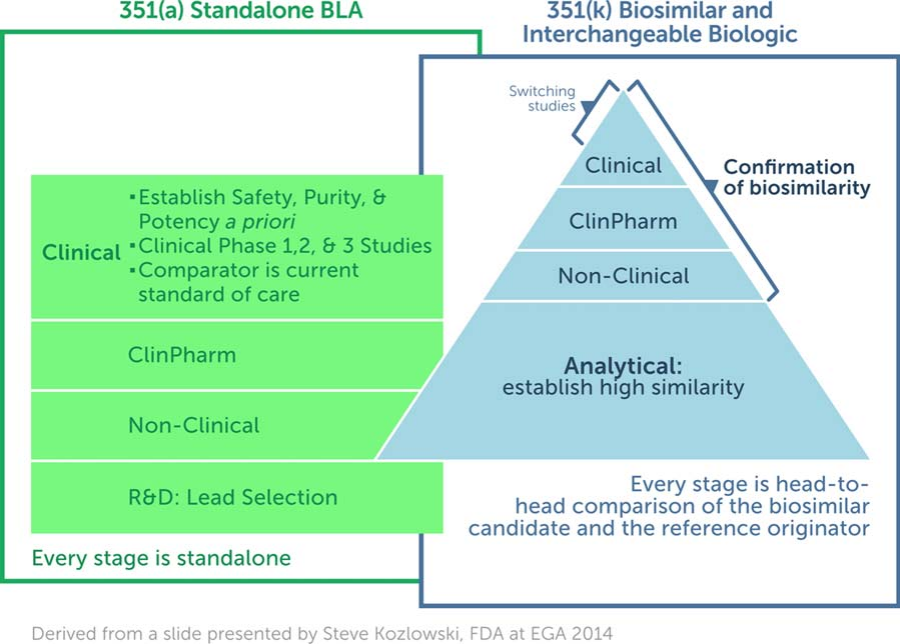
A representation of the data requirements for the development of a standalone biologic [351(a)] as compared to those expected for a biosimilar/interchangeable biologic [351(k)]. The concept of biosimilarity is fundamentally different from that applied to an originator biologic where safety, purity, and potency must be established *a priori*.

**Figure** 1 illustrates how a standalone biologic requires initial investment in research and development to determine the lead candidate and then extensive clinical development, as opposed to the substantially greater investment in analytics for a biosimilar and a reduced clinical data package to confirm biosimilarity.

### Clinical studies confirm biosimilarity, they do not establish safety, purity, and potency *a priori*

As such, clinical studies on a biosimilar are not designed in the same manner as those for an originator biologic, where safety, purity, and potency are being established for the first time. The biosimilar relies on the known clinical outcomes for the originator and are designed to reduce any residual uncertainty left after the completion of the head‐to‐head analytical comparison. They are conducted in a sensitive population and scaled appropriately. When the mechanism of action is well understood, the biosimilar sponsor can show a match for one, some, or all of the indications of the reference product. An unknown mechanism of action still allows for extrapolation just as occurs during comparability. Extrapolation to all indications of the reference product is NOT an extrapolation from the single clinical study of the biosimilar to those other indications. Extrapolation relies on demonstrating biosimilarity, and any clinical study provides the final data component confirming similarity (“sameness” of the active pharmaceutical ingredient). When similarity is confirmed, then the extrapolation is to the reference product that has already established safety and efficacy in each of those multiple indications.

For biosimilar development, abbreviation in the clinical development program is essential and an expectation of extrapolation is integral to the selection of the reference product. This is how greater access and affordability through biosimilars will be possible in the United States, as already occurs in Europe. There is no compromise in the final quality of the biosimilar, nor of its suitability for any indication for which it is labeled. However, focused development only on those studies that are necessary to confirm biosimilarity must occur.

**Figure 2:**
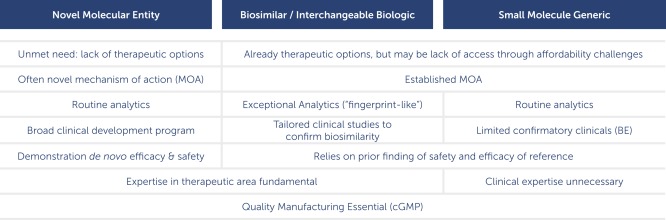
A comparison of the regulatory expectations for a novel molecular entity (drug or biologic), for a classic small molecule generic, and for a biosimilar/interchangeable biologic. The latter shares attributes of the other two.

**Figure** 2 shows a comparison of those attributes of a standalone innovator product that contains a novel active ingredient compared to a biosimilar that contains the “same” active pharmaceutical ingredient and also to a generic small molecule drug, which has an identical active pharmaceutical ingredient.

### Interchangeability is inevitable for a generic drug or for a post‐manufacturing change biologic using comparability

However, as a legal matter, the Biologics Price Competition and Innovation Act requires a biosimilar sponsor to demonstrate that switching between the interchangeable biologic and its reference product during a course of treatment does not create a greater risk for the patient (defined as loss of efficacy or a safety issue). A designation of interchangeability allows the interchangeable biologic to be substituted for its reference by the pharmacist in the manner of a small molecule generic drug (no prior permission from the original prescriber being necessary).

A sponsor can elect to apply for an interchangeable designation as part of their initial approval, but this is considered unlikely given that the FDA has expressed preference for a two‐step approach (first gain biosimilarity, then apply for interchangeability). Although formal guidance is not available, it is assumed that, in a two‐step development plan, clinical studies switching the same patients between the reference product and the already‐licensed biosimilar will be compared to patients that are not switched, and the similarity of the clinical outcomes in both populations thereby confirmed.

The decision to pursue an FDA designation of interchangeability will be determined by the resources required to do the switching studies, as well as their scientific feasibility^5^; and these will be considered in the context of the commercial outcome expected. However, the product on which the switching studies are conducted will be the same as that designated as a biosimilar (already or concurrently) established by in‐depth analytics and limited confirmatory clinical studies.

## CONCLUSIONS

Biosimilars are designed to match their reference product as closely as is scientifically possible from day one of development. The interchangeable biologic candidate on which the switching studies are conducted is identical to the biosimilar approved without an interchangeability designation. There is no higher regulatory standard but there is greater data burden to obtain an interchangeability designation.

The decision to pursue a formal FDA designation of interchangeability will be governed by the willingness of the FDA to make such a determination with an economically feasible clinical data package in addition to the commercial value of such a designation. The FDA has experience in approving complex products as interchangeable using established scientific criteria for such an approval, as was seen with the approval of the complex generic of enoxaparin, and for manufacturing changes. Ultimately, patient benefits will be determined by access to biosimilars, and patients will not benefit from unnecessary clinical studies being proposed. Not least given that the interchangeable biologic is simply a biosimilar on which additional studies have been conducted.

## CONFLICT OF INTEREST

M.McC., J.P., and C.S. are employees of Sandoz and G.W. is an employee of Avalere Health.
